# R language-based methods for revealing taxonomic and functional diversity and tracing process of fish fauna

**DOI:** 10.1016/j.mex.2018.11.003

**Published:** 2018-11-07

**Authors:** Bin Kang, Xiaoxia Huang, Yunzhi Yan, Yunrong Yan, Hungdu Lin

**Affiliations:** a College of Fisheries, Ocean University of China, Qingdao, 266003, China; b Key Laboratory of Atmospheric Environment and Processes in the Boundary Layer Over the Low-latitude Plateau Region, School of Earth Science, Yunnan University, Kunming, 650091, China; c College of Life Sciences, Anhui Normal University, Wuhu, 241002, China; d College of Fisheries, Guangdong Ocean University, Zhanjiang, 524088, China

**Keywords:** GIS, R language, Regression analysis, Species richness, Taxonomic diversity, Functional diversity, Species turnover/nestedness, Conservation

## Abstract

This paper presented the fish species richness at geographical unit of the Yangtze River. According to the fish taxonomic catalogs and biological traits, R language method was used to determine taxonomic diversity and functional diversity and the components of each unit. Regression analysis was used to test the varying tendency of taxonomic and functional diversity corresponding to the change of species richness.

• Functional diversity is compared against taxonomic diversity in capturing the structure of dynamic ecosystem.
• The β-diversity indices of taxonomy and function were calculated and decomposed to evaluate the role of species turnover and nestedness in the formation process of fish spatial pattern.
• An integrated diversity index, balancing α and β diversity of species richness, taxonomic and functional diversity, is used to screen the prior conservation zone.

## Graphical abstract

The figure showed the spatial pattern of an integrated index of multifaceted diversity in the Yangtze River Basin, considering the higher fish species richness and functional contribution. Headwater (characterized by abundant endemic species), the middle mainstream (necessary to fish migration), and the lakes (showing higher diverse species) were primary zones for conservation.

**Figure:**
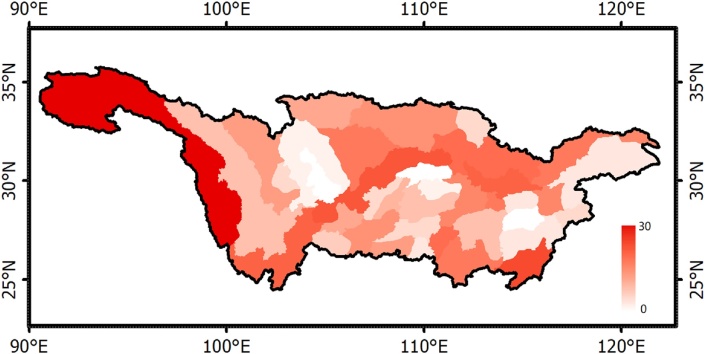

**Specifications Table**

**Table:** 

**Subject Area**	• *Agricultural and Biological Sciences*
**More specific subject area:**	*Biodiversity and conservation, fisheries*
**Method name:**	*GIS, R language, Regression analysis*
**Name and reference of original method**	*Baselga, A., 2012. The relationship between species replacement and dissimilarity derived from turnover and nestedness. Glob. Ecol. Biogeogr. 9, 134-143.* *Casanoves, F., Pla, L., Rienzo, J.A.D, Díaz, S. (2011) FDiversity: a software package for the integrated analysis of functional diversity. Methods Ecol. Evol., 2, 233-237.* *Clarke, K.R., Warwick, R.M. (1998) A taxonomic distinctness index and its statistical properties. J. Appl. Ecol., 35, 523-531.*
**Resource availability**	*Fishbase:*http://www.fishbase.org/search.php *Shuttle Radar Topography Mission database:*https://lta.cr.usgs.gov/SRTM,

## Method details

### Geological parameters

To determine the spatial pattern process of fish diversity at taxonomic and functional aspects concerning to the environmental characters, we divided the Yangtze River Basin could be into 11 sub-basins as Jinsha, Min-Tuo, Jialing, Han, Wu, the upper mainstem, the middle mainstem, the lower mainstem, Dongting, Poyang, and further into 56 geographic units, according to the natural river system and annual discharge.

The longitude, latitude, altitude, and channel length data of each unit were derived through the Spatial Analyst Tool in ArcGIS 10.0 [1]. The longitude and latitude extents of a unit were represented by a rectangle envelope spanning the minimum and maximum *x*, *y* coordinates of the region, the four points and the centre of gravity of an envelope enclosing the unit were computed using the toolset. At the end, the maximum, minimum and mean values of each factor were calculated by comparing and averaging the values of each grid within the sub-basin through the Zonal Statistics tool of Spatial Analyst. The altitude data with a spatial resolution of 3 arc-seconds (∼90 meters) was provided by the Shuttle Radar Topography Mission database (https://lta.cr.usgs.gov/SRTM) download from the International Scientific & Technical Data Mirror Site (Computer Network Information Centre, Chinese Academy of Sciences, http://datamirror.csdb.cn).

The hydrologic analysis in Spatial Analyst Toolbox of ArcGIS was used to model the movement of water across a basin. An eight-direction (D8) flow model [2] was used to determine the direction of flow (a direction of steepest descent) from every cell in the elevation raster. After creating a hydrologically conditioned elevation model (the sinks were identified and filled) from a digital elevation model (DEM), the flow accumulation for each cell location could be calculated. Supposing when enough water flows through a cell, there should be a stream passing through it, thus defining the stream network. The length of all streams combine in a unit was summed as channel lengths.

### Taxonomic diversity approach

Taxonomic diversity (TD), incorporating the degree to which the species are taxonomically related to each other, can be used to describe biological diversity of an area more than the numbers of species present. For example, TD of an assemblage containing *n* species belonging to *x* genus is larger than that of another assemblage containing *n* species belonging to *y* genus (*x*>*y*). The Average Taxonomic Distinctness (AvTD), defined as the average taxonomic path length, was calculated based on the taxonomic distance through a classification tree at four taxonomic levels (species, genus, family, order) between every pair of species, with an advantage of not being affected by the number of species or the sampling efforts [3]. The AvTD (Δ+) is determined from data consisting only of presence or absence of taxa:

Δ+ = [∑∑*_i<j_ω_ij_*]/[*s*(*s*-1)/2)];

Total Taxonomic Diversity (TTD, *s*Δ+) sums up distinctiveness values for particular species across the community:

*s*Δ+ =∑*_i_*[(∑*_j≠i_ ω_ij_*)/ (*s*-1)],

where *s* is the number of species present, the double summation is over the set {*i* = 1,…*s*; *j* = 1,…*s*, such that *i < j*}, and *ωij* is the ‘distinctness weight’ between species *i* and *j*.

All the taxonomic diversity indices calculation was undertaken using R language [4] (Appendix A in Supplementary materials).

### Functional diversity indices

Functional richness, representing the amount of functional space filled by a community, quantifies the functional diversity for unit with species distributed in a multidimensional functional space [5]. The relationship between taxonomic and functional diversity suggests a predominant influence of environmental filters on the functional structure of communities, which is not necessarily reflected in their taxonomic structure. As probably several species showed similar functional traits, FD variation was small than SR. Similarly, as several species with differentiated functional traits belonged to the same taxon, TD variation was small than FD.

In order to estimate the volume filled in the T dimensional space by the community of interest, the quick hull algorithm was used to compute the minimum convex hull including all considered species.

First, a functional distance matrix based on biological traits of each species was computed, using Gower’s distance, to compile a metric able to accommodate nominal, ordinal, continuous and missing data. Then, a Principal Coordinates Analysis (PCoA) was constructed to represent the multivariate traits space occupied by the fishes. Following a trade-off between information quality and computation time, we finally kept the species coordinates on the first three axes as the values of three synthetic functional traits describing fish functional strategies. All calculations of functional diversity indices were performed in the R statistical and programming environment using the ‘FD’ and ‘vegan’ packages [4] (Appendix B in Supplementary materials).

### The β-diversity and its components

The pairwise β-diversity (species richness and taxonomic diversity) of units’ fish communities was measured using the Jaccard dissimilarity index (*β_jac_*) based on the number of species, and divided into β-diversity turnover (*β_jtu_*) and β-diversity nestedness (*β_jne_*) [6]. The *β_jtu_* measures the proportion of species that would be replaced between assemblages if both assemblages had the same number of species, hence accounts for species replacement without the influence of richness differences; *β_jne_* reflects the increasing dissimilarity between nested assemblages resulting from the increasing differences in species richness. The calculation formulas were:

*β_jac_* = *β_jtu_* + *β_jne_*,

*β_jac_* = (*b* + *c*)/(*a* + *b* + *c*),

*β_jtu_* = 2 min (*b* + *c*)/(a + 2 min (*b*, *c*),

*β_jne_* = {[max (*b*, *c*) - min (*b*, *c*)]/(*a* + *b* + *c*)}*{*a*/[*a* + 2 min (*b*, *c*)]},

Where *a* is the number of species present in both sites, *b* is the number of species present in the first site but not in the second, and *c* is the number of species present in the second site but not in the first.

Based on the convex hull volume, functional β-diversity = (functional space not shared) / (total functional space filled) was decomposed into functional turnover and functional nestedness components as:

Functional β-diversity = [V(C1)+V(C2)-2 V(C1∩C2)]/[V(C1) + V(C2)-V(C1∩C2)],

Functional turnover = [2 min(V(C1), V(C2)) - 2V(C1∩C2)]/[2 min(V(C1), V(C2)) - V(C1∩C2)],

Functional nestedness = {│V(C1) -V(C2)/[V(C1) + V(C2)-V(C1∩C2)]}*{V(C1∩C2)/[2 min(V(C1), V(C2)) - V(C1∩C2)]},

Where V(C1) and V(C2) are the volume of the convex hulls of each of the two communities(C1 and C2), V(C1∩C2) is their intersection.

According to equation of *β_jac_*,

a = V(C1∩C2), b = V(C1) - V(C1∩C2), and c = V(C2) - V(C1∩C2).

For each unit, we calculated the mean β-diversity by computing the average of the pairwise comparisons between each focal basin and all the remaining basins. All calculations of functional diversity indices were performed in the R statistical and programming environment using the ‘FD’, ‘betapart’, and ‘vegan’ packages [4] (Appendix C in Supplementary materials).

### Integrated diversity index

The 9 aspects of calculated diversity indices (species richness, β-species richness turnover, β-species richness nestedness, taxonomic diversity, β-taxonomic diversity turnover, β-taxonomic diversity nestedness, functional diversity, β-functional diversity turnover, β-functional diversity nestedness), each reflecting an aspect of fish fauna, were normalized to eliminate the gaps of original numerical differences：

y_i_ = [x_i_−min(x_j_)]/[max(x_j_)−min(x_j_)], (1 ≤ i ≤ n, 1 ≤ j ≤ n),

where x_i_ is the original data, max(x_j_) and min(x_j_) are the maximum and minimum values of the sample data, respectively, and y_i_ is the normalized data.

A trade-off integrated index was determined by summing all the normalize values, and then sorted to screen the prior zones for conservation;

$$\text{IDI}=\Sigma_{\text{i}=1}^{9}\text{y}\text{i},$$

where i means different aspect of diversity.
