# Supplementary material for: R language-based methods for revealing taxonomic and functional diversity and tracing process of fish fauna
Source: MethodsX. 2018 Nov 7;5:1498–502. doi: 10.1016/j.mex.2018.11.003 (PMC6251016; doi:10.1016/j.mex.2018.11.003)
Supplement: Supplementary file 1 [file mmc1.docx]

**Supplementary material *and/or* Additional information:**

**Appendix A. R codes for calculating taxonomic diversity index**

# Set Working Directory

setwd(“file directory”)

# Load the package

library(vegan)

# Read the data file

fish<-read.csv("filename ",header=T, row.names=1,sep=",")

tax<-read.csv("filename ",header=T, row.names=1, sep=",")

#Transform the dataframe for diversity calculation
#(369 fish * 56 basin transform to 56 basin*369 fish)

t_fish<-t(fish)

# Taxonomic distances from a classification table with variable step lengths

taxdis <- taxa2dist(tax, varstep=TRUE)

# Calculate taxonomic diversity and distinctness

mod <- taxondive(t_fish, taxdis)

# List the result

mod

**Appendix B. R codes for calculating functional diversity index**

# Set Working Directory

setwd(“file directory”)

# Load the package

library(vegan)

library(FD)

# Read the data file

fish<-read.csv("filename ",header=T, row.names=1,sep=",")

tax<-read.csv("filename ",header=T, row.names=1, sep=",")

trait<-read.csv("filename ",header=T, row.names=1, sep=",")

#Prepare trait data for FRic calculation

gower.f<-gowdis(trait) # Gower’s distance analysis

pcoa.g<-pcoa(gower.f,rn=NULL ) # PCoA analysis

Fun_pcoa<-pcoa.g$vectors[,1:3] # the first three axes of PCoA were kept as

# the synthetic functional traits

#Transform the dataframe for diversity calculation
t_fish<-t(fish) #(369 fish * 56 basin transform to 56 basin*369 fish)

# Calculate functional diversity indices

FD.alpha<-dbFD(Fun_pcoa, t_fish)

# List the result

FD.alpha$FRic

**Appendix C. R codes for calculating β-diversity index, and its component**

# Set Working Directory

setwd(“file directory”)

setwd("file directory ")

# Load the packages

library(vegan)

library(FD)

library(betapart)

# Read the data file

fish<-read.csv("filename ",header=T, row.names=1,sep=",")

tax<-read.csv("filename ",header=T, row.names=1, sep=",")

trait<-read.csv("filename ",header=T, row.names=1, sep=",")

#Prepare trait data for calculation

gower.f<-gowdis(trait) # Gower’s distance analysis

pcoa.f<-pcoa(gower.f,rn=NULL ) # PCoA analysis

Fun_pcoa<-pcoa.f$vectors[,1:3] # keep the first three axes of PCoA as

# the synthetic functional traits

#Prepare taxonomic data for calculation

gower.t<-gowdis(tax,ord = c("classic")) # Gower’s distance analysis

pcoa.t<-pcoa(gower.t,rn=NULL ) # PCoA analysis

tax_pcoa<-pcoa.t$vectors[,1:3] # the first three axes of PCoA were kept as

# the synthetic taxonomic traits

#Transform the dataframe for diversity calculation

t_fish<-t(fish) #(N fish * M basin transform to M basin*N fish)

#Analysis the beta indice between sub-basins

SR_beta<- beta.pair(t_fish, index.family="jaccard")

FD_beta<-functional.beta.pair(t_fish, Fun_pcoa, index.family="jaccard")

TD_beta<-functional.beta.pair(t_fish, tax_pcoa, index.family="jaccard")

#Get the results

SR.jtu<-as.matrix(SR_beta$beta.jtu)

SR.jne<-as.matrix(SR_beta$beta.jne)

SR.jac<-as.matrix(SR_beta$beta.jac)

FD.jtu<-as.matrix(FD_beta$funct.beta.jtu)

FD.jne<-as.matrix(FD_beta$funct.beta.jne)

FD.jac<-as.matrix(FD_beta$funct.beta.jac)

TD.jtu<-as.matrix(TD_beta$funct.beta.jtu)

TD.jne<-as.matrix(TD_beta$funct.beta.jne)

TD.jac<-as.matrix(TD_beta$funct.beta.jac)

#average beta between sub-basins, for M sub-basins, the summary should be divided by (M-1).

Beta_bs<-cbind(colSums(FD.jac)/ (M-1), colSums(FD.jtu)/ (M-1), colSums(FD.jne)/ (M-1),
colSums(TD.jac)/ (M-1), colSums(TD.jtu)/ (M-1), colSums(TD.jne)/ (M-1),
colSums(SR.jac)/ (M-1), colSums(SR.jtu)/ (M-1), colSums(SR.jne)/ (M-1))

colnames(Beta_bs)=c('avg_FD.jac', 'avg_FD.jtu', 'avg_FD.jne', 'avg_TD.jac', 'avg_TD.jtu', 'avg_TD.jne',
'avg_SR.jac', 'avg_SR.jtu', 'avg_SR.jne')

Beta_bs

# Load the package

library(pheatmap)

# Heatmap of beta-diversity between sub-basins

pheatmap(FD.jtu,main="FD.jtu")

pheatmap(FD.jne,main="FD.jne")

pheatmap(FD.jac,main="FD.jac")

pheatmap(TD.jtu,main="TD.jtu")

pheatmap(TD.jne,main="TD.jne")

pheatmap(TD.jac,main="TD.jac")

pheatmap(SR.jtu,main="SR.jtu")

pheatmap(SR.jne,main="SR.jne")

pheatmap(SR.jac,main="SR.jac")

#Calculate the proportion of turnover and nestedness components in the overall dissimilarity
#of functional beta diversity and taxonomic beta diversity

B1<- FD.jtu*FD.jac^-1

B2<- FD.jne*FD.jac^-1

B2[lower.tri(B2)]=0

B1[upper.tri(B1)]=0

FD_beta.r<- B1+B2

pheatmap(FD_beta.r, cluster_rows = F, cluster_cols = F, show_rownames = T, show_colnames = T)

B1<- TD.jtu*TD.jac^-1

B2<- TD.jne*TD.jac^-1

B2[lower.tri(B2)]=0

B1[upper.tri(B1)]=0

TD_beta.r<- B1+B2

pheatmap(TD_beta.r, cluster_rows = F, cluster_cols = F, show_rownames= T, show_colnames = T)

B1<- SR.jtu*SR.jac^-1

B2<- SR.jne*SR.jac^-1

B2[lower.tri(B2)]=0

B1[upper.tri(B1)]=0

SR_beta.r<- B1+B2

pheatmap(SR_beta.r, cluster_rows = F, cluster_cols = F, show_rownames= T, show_colnames = T)
